# The role of macrophages in cardiac diseases: a review of the origins, functional diversity, and therapeutic potential

**DOI:** 10.3389/fimmu.2026.1896304

**Published:** 2026-09-14

**Authors:** Li Che, Zilong Wang

**Affiliations:** 1 Department of Cardiology, Central Hospital of Dalian University of Technology, Dalian, China; 2 Department of Neurosurgery, The First People’s Hospital of JinZhou District, Dalian, China

**Keywords:** cardiac diseases, immune regulation, macrophages, metabolic reprogramming, therapeutic strategies

## Abstract

Macrophages are important immune cells for cardiac development, homeostasis, and the response to pathological injury and inflammation. Single-cell sequencing and fate-mapping technologies have revealed the heterogeneity and development of cardiac macrophages. They are classed into two main groups: embryonic tissue-resident and bone marrow-derived macrophages. These different macrophage subsets show complex functions in cardiac diseases such as inflammation regulation, tissue repair, metabolic reprogramming and intercellular communication within the cardiac microenvironment. This review discusses the developmental sources, phenotypic diversity, metabolic features and mechanistic functions of cardiac macrophages in different cardiac disorders. We focus on macrophage polarization, metabolic regulation during disease progression and targeted strategies and future research directions.

## 1 Introduction

The complex interactions that occur between macrophages and cardiac diseases have attracted considerable research interest in recent years (1, 2). More specifically, macrophages are important components of the innate immune system, maintaining cardiac homeostasis and coordinating repair following myocardial infarction (MI) (3). The functions of macrophages also include simple phagocytosis, mediation of inflammatory responses, tissue remodeling, and cardiac function regulation (4). Hence, understanding the heterogeneity of macrophages in cardiac diseases is important due to their different origins, phenotypes, and biological functions.

Recent progress in relation to single-cell sequencing has highlighted the heterogeneity of cardiac macrophages, which has in turn revealed their differing embryonic and hematopoietic origins. In fact, there are two subsets of cardiac macrophages—namely, resident and infiltrating macrophages. The former mainly originate from yolk sac progenitors, whereas the latter stem from circulating monocytes (5). Macrophages exhibit changes following MI, shifting from pro-inflammatory (M1) states to reparative (M2) states, which is essential for both cardiac repair and recovery (5). The M1/M2 dichotomy is merely a simplified model. The actual state of macrophages presents a continuous lineage and is dynamically regulated by the microenvironment.

Historically, metabolic reprogramming was considered to be important in terms of regulating macrophage polarization and function. In particular, research focused on how M1 states mainly depend on glycolysis and produce pro-inflammatory cytokines, while M2 states depend on oxidative phosphorylation and aid in tissue repair (6). Thus, an imbalance between these two states may result in inflammation and cardiac remodeling (7). However, this is now regarded as an oversimplification of the biology of cardiac macrophages *in vivo*. Indeed, recent single-cell transcriptomics studies have revealed a continuous spectrum of activation states beyond the initial binary classification, which are defined by markers such as CCR2, LYVE1, and MHCII (8–10). This review discusses macrophage function during specific disease stages, in different microenvironments, and among molecular subtypes other than the M1/M2 frameworks.

Macrophages are known to interact with other types of cells (e.g., cardiomyocytes, fibroblasts, endothelial cells) (11). Different signaling pathways and cytokines participate in these interactions, which both modulate the local microenvironment and impact the response to injury. Macrophages secrete factors in order to increase angiogenesis and regulate fibroblast function (12). As a consequence, macrophages play a complex role in heart disease, and their different origins, phenotypic diversity, and varying interactions with other cardiac cells emphasize the complexity of their functions. This suggests the possibility of new means of treatment to promote cardiac repair, rendering macrophage-targeted strategies a promising avenue for future cardiac disease treatments.

In light of this, the present review focuses on the role of macrophages in heart diseases, including MI, heart failure, myocarditis, fibrosis, and arrhythmia. However, it does not examine the role of macrophages in vascular inflammation, hypertension-related vascular remodeling, or peripheral vascular disease.

## 2 Origin and classification of cardiac macrophages

### 2.1 Embryonic-derived tissue-resident macrophages

Embryonic-derived TRMs are important in relation to cardiac development and homeostasis. These macrophages emerge from the yolk sac during early embryonic development. They represent one of the first types of immune cells to regulate the heart and can persist in the long term and maintain self-renewal throughout their life (13). These TRMs differ from the monocyte-derived macrophages that infiltrate tissues during inflammation, and they are integral components of the cardiac structure and function from the developmental stage. They participate in important processes through regulating local immune reactions and clearing apoptotic cells via efferocytosis (14). The unique transcriptional signatures and surface markers of embryonic-derived TRMs (particularly TIMD4^+^ and CCR2^-^) indicate their function in both tissue repair and immune regulation, which differentiates them from monocyte-derived macrophages with typical pro-inflammatory traits (15).

Single-cell RNA sequencing (scRNA-seq) studies have revealed the functional diversity of cardiac macrophages and demonstrated that embryonic-derived TRMs are important when it comes to the heart’s regenerative capacity following injury (16). These macrophages support cardiac structural integrity and facilitate communication between immune cells and cardiomyocytes, thereby regulating cardiac function and the response to stress (17). The role of embryonic-derived TRMs in the early stages of heart development is critical—that is, they shape the cardiac microenvironment, promote vascular formation, and support electrical conduction pathways (18). Studies have shown that macrophage loss during developmental periods can result in significant cardiac defects, which emphasizes their important role in heart development (19).

Moreover, the functional plasticity of embryonic-derived TRMs allows them to adapt to various physiological and pathological states. For example, in response to cardiac damage, these macrophages shift from playing a homeostatic role to adopting a reparative state, which can aid in inflammation resolution and promote tissue repair (20). This adaptability is supported by the ability of TRMs to shape local cytokine environments, which is crucial for directing repair processes following cardiac damage (21). Embryonic-derived TRMs are passive residents and can both maintain cardiac health and respond to injury. Their unique origins and functions suggest their utility in targeted therapeutic treatments. Thus, enhancing TRM activity or replicating their reparative functions in heart diseases could provide new means of treatment for such diseases.

### 2.2 Bone marrow-derived monocyte-derived macrophages

Bone-marrow-derived MDMs play an important role in the pathological processes of cardiac diseases, especially during cardiac injury and inflammation. These macrophages originate from the hematopoietic system in adult bone marrow and are mobilized to the heart in response to triggers such as tissue damage or inflammatory signals (22). Activated monocytes express the chemokine receptor CCR2, which is key to their migration to inflamed tissues, including the heart (23). Cell recruitment is significantly increased in the event of cardiac disorders (e.g., MI, heart failure), wherein bone-marrow-derived MDMs participate in the inflammatory response and tissue repair. Their activity in the heart is passive, including when regulating inflammatory responses, affecting cardiac diseases via their diverse functions, such as pro-inflammatory cytokine secretion and cell debris phagocytosis (24).

Monocytes are classified into three subgroups—namely, classical monocytes (CD14++CD16-), intermediate monocytes (CD14++CD16+), and non-classical monocytes (CD14+CD16++) (25). The expansion of intermediate monocytes (CD14++CD16+) in the bloodstream is associated with an increased risk of adverse cardiac events and heart failure following acute MI (26). Moreover, changes in non-classical monocytes (CD14+CD16++) in patients with heart failure are associated with the severity and prognosis of the disease, although their specific directions of action have been found to vary across studies (27). In addition, the proportion of classical monocytes (CD14++CD16-) was determined to decrease in patients with acute decompensated heart failure, although it rebounded after treatment, suggesting that they may be involved in the process of inflammation resolution (28). Together, these findings indicate that different monocyte subsets play complex and possibly even contradictory roles in the occurrence and development of heart diseases.

Bone-marrow-derived MDMs exhibit functional diversity due to their ability to adopt different polarization states, which are primarily divided into the pro-inflammatory and reparative subtypes. Maintaining a balance between these two states is important for sustaining cardiac homeostasis and determining disease outcomes. Different signals, such as cytokines and metabolic cues, drive this functional plasticity and can alter the macrophage phenotype, thereby impacting MDMs’ role in cardiac pathology (29).

Additionally, the recruitment and activation of bone-marrow-derived MDMs are associated with cardiac diseases. For instance, studies have shown that the number of these macrophages correlates with the severity of both cardiac injury and inflammation. Moreover, the changes that MDMs undergo in response to cardiac stressors give rise to their role as contributors and responders to the pathological state of the heart (30).

It is recognized that cardiac macrophages also originate from embryonic sources other than yolk sac progenitor cells (31). For example, monocytes derived from the fetal liver contribute to the initial pool of cardiac-resident macrophages, and their relative proportion varies with age (14, 32). Even during homeostasis, resident macrophages may be partially replaced by circulating monocytes. This process is mediated by two pathways—namely, the CCR2-dependent and CCR2-independent pathways—which generate different lineages. For instance, CCR2^-^ macrophages typically possess self-sustaining capabilities and are tissue-resident, whereas CCR2^+^ macrophages, which originate from monocytes, are rapidly recruited during inflammation (10). Understanding the dynamic changes that occur in these lineages is crucial to analyzing their different roles in cardiac homeostasis and disease.

### 2.3 Molecular markers and functional differences of macrophage subpopulations

ScRNA-seq has advanced rapidly in recent years, reshaping understanding of the different functional states and cellular communities of macrophages. In particular, this high-resolution technique helps identify separate macrophage subpopulations. Each subpopulation displays a specific profile that is associated with specific functions in terms of health and disease. Studies have shown that macrophages can be classified into different states according to their gene expression patterns, which can reflect their activation status and functional roles (33). In cardiac diseases, these subpopulations can typically be defined as pro-inflammatory or reparative, although there are also intermediate states that exhibit hybrid characteristics (34).

The classical model distinguishes pro-inflammatory or reparative macrophages, but it has been updated to include many subtypes. New markers such as SPP1 and IL1B can identify the specific macrophage groups that are associated with inflammation and tissue repair (35). These markers therefore help classify macrophages and facilitate a deeper understanding of their functions. More specifically, SPP1+ macrophages are associated with fibrotic responses, while IL1B+ macrophages are associated with acute inflammation (36).

Yet the markers present in cardiac macrophages and their specific subsets remain to be elucidated. Recent studies have identified subsets of cardiac macrophage markers, such as CCR2, MHC-II, and Ly6C, which demonstrate different functions in the maintenance of homeostasis, inflammation, and repair (37, 38). Further exploration is required to determine whether SPP1+ and IL1B+ subpopulations exist in the heart and identify their relation with disease progression.

## 3 Metabolic reprogramming and functional regulation of macrophages

### 3.1 the impact of metabolic state on macrophage function

The metabolic state of macrophages is crucial when it comes to regulation of functional responses, which is mainly controlled by the balance between glycolysis and oxidative phosphorylation. This metabolic flexibility enables macrophages to adapt their energy production pathways according to the stimulation and environmental conditions. Macrophages polarize into different phenotypes, with each type showing a specific metabolic profile. This metabolic reprogramming represents a response to the local environment and is regulated by different signaling pathways. These pathways combine information on nutrient and cellular energy levels in the body to regulate macrophage activation and polarization (36).

TRMs are important in relation to homeostasis and usually rely on oxidative phosphorylation, which enables them to regulate local tissue environments and respond to homeostatic signals while avoiding excessive inflammation. In fact, resident macrophages use oxidative phosphorylation to maintain and repair cardiac tissue, and this helps prevent pathological remodeling under stress conditions such as ischemia (39). In contrast, MDMs often increase their glycolytic activity during inflammation, and this shift is key to supporting the elevated energy requirements of inflammatory responses. In particular, it supports the production of inflammatory cytokines and reactive oxygen species (39). This metabolic shift is apparent following acute MI, where the infiltrating macrophages change from a pro-inflammatory state to a reparative state, a temporal metabolic adaptation that is critical for tissue repair.

### 3.2 The role of metabolites in cardiac macrophage signaling

Key metabolites such as lactate and succinate can regulate macrophage function, especially under ischemic conditions. Lactate was once regarded as a byproduct of anaerobic metabolism, although it is now recognized as a signaling molecule. This molecule can induce macrophage polarization to shift to a reparative state that may increase reparative cytokine production, promoting tissue repair and regeneration following cardiac injury. Indeed, lactate can increase the presence of reparative cytokines in macrophages, which helps reduce the inflammatory response that worsens cardiac damage after MI (40). Moreover, succinate is a key intermediate in the tricarboxylic acid (TCA) cycle and can activate the succinate receptor GPR91. This process both affects macrophage polarization and drives pro-inflammatory responses (41).

The regulation of metabolic pathways is key to the fate of monocytes and the shaping of cardiac macrophage subtypes, which is linked to their function. Macrophages can drive metabolic reprogramming and affect the inflammatory profile under hypoxic conditions. This function is mediated by hypoxia-inducible factors, which induce a shift from oxidative phosphorylation to glycolysis, thereby increasing the production of pro-inflammatory mediators (42).

### 3.3 Metabolic regulation and its connection to cardiac diseases

As key immune cells, macrophages can change metabolic profiles in an effort to support their pro-inflammatory functions, leading to cardiac damage. Studies indicate that macrophages can switch metabolic pathways from oxidative phosphorylation to glycolysis during inflammation, which supports their survival and function but may lead to increased tissue damage (43). This metabolic shift is not just a byproduct of inflammation; rather, is closely linked to macrophages’ ability to produce inflammatory cytokines and mediators that can damage the heart. Furthermore, the interaction that occurs between metabolic disorders and immune activation in macrophages reveals the complex relation between systemic diseases and cardiac health.

Moreover, identifying metabolic targets in macrophages represents a promising strategy for immunotherapy in patients with cardiac diseases. Recent research has focused on macrophage metabolism in an attempt to reduce inflammation and promote heart tissue repair. Interventions that promote a shift from pro-inflammatory phenotypes to reparative macrophage phenotypes via metabolic reprogramming have demonstrated potential in terms of reducing cardiac fibrosis and improving heart function (43). Thus, taking advantage of macrophages’ plasticity and their metabolic pathways could lead to new immunomodulatory therapies.

The opposite effects of lactic acid and succinic acid in ischemic hearts illustrate a key conceptual gap—that is, while both are elevated under hypoxic conditions, lactic acid promotes reparative polarization, whereas succinic acid drives pro-inflammatory responses. The net effect of these functions on tissue repair and fibrosis depends on the relative concentrations, spatial distribution, and temporal dynamics. Future studies should explore how the balance between these metabolic signals determines the fate of macrophages and how best therapeutic management could be performed (**Figure 1**).

**Figure 1:**
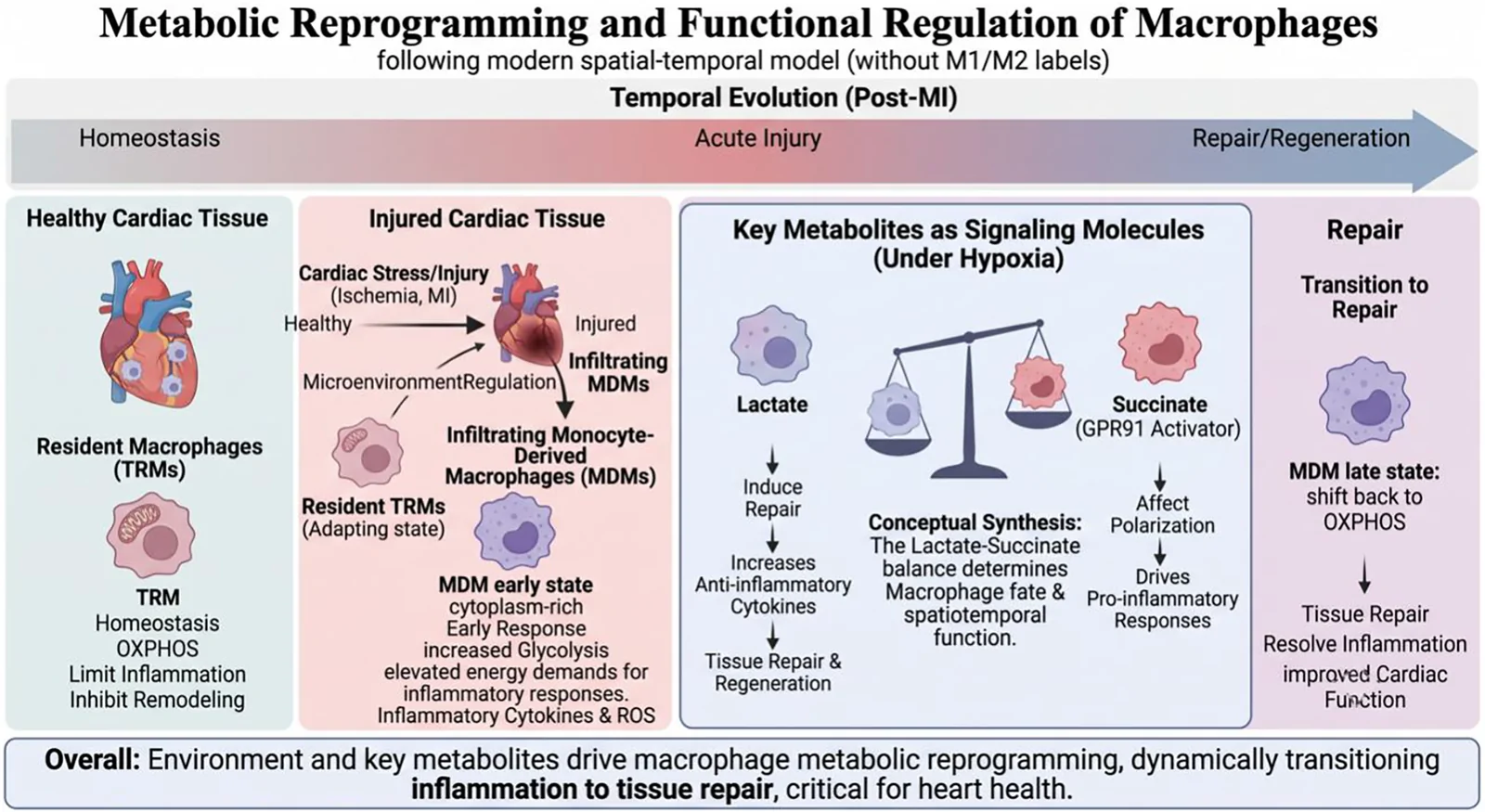
Metabolic reprogramming and functional regulation of macrophages.

## 4 The specific mechanisms of macrophages in cardiac diseases

### 4.1 Macrophages in myocardial infarction and ischemic heart disease

Recent single-cell transcriptomics studies have deepened understanding of the heterogeneity of cardiac macrophages beyond the simple binary classification of activation states. Specific markers, such as TIMD4, LYVE1, and MHCII, have been used to define different subgroups with unique functions. For instance, LYVE1^+^ macrophages are associated with tissue repair and angiogenesis, while MHCII^+^ macrophages are related to antigen presentation and immune regulation (8, 9). Together, these markers show a continuous spectrum of the activation state, albeit not discrete categories.

During the late stage of MI, pre-existing resident macrophages (CCR2^-^, embryonically-derived) play different roles, promoting angiogenesis and tissue repair but having only a limited impact on excessive fibrosis. However, recruited monocyte-derived macrophages (CCR2^+^) migrate to the infarcted area in response to chemokines released by damaged cardiomyocytes and other cells. Their main functions during this phase include phagocytosis of necrotic cells and debris, in addition to secretion of pro-inflammatory cytokines that enhance local inflammatory responses. This inflammatory response is necessary for clearing dead cells and promoting the healing process, although excessive activation can worsen MI. In fact, inflammatory macrophages can lead to adverse remodeling and impair cardiac function, which indicates the importance of maintaining a balance between the protective and harmful effects (24).

Macrophages change from pro-inflammatory to reparative phenotypes during the healing process, which is important for tissue repair and regeneration. During the late stages of MI, resident macrophages are important in relation to inflammation and promotion of both angiogenesis and fibrosis. More specifically, they secrete growth factors and cytokines to promote development of new blood vessels and drive extracellular matrix (ECM) deposition. Of course, excessive fibrosis can lead to stiffness and weaken contraction, while poor repair may lead to heart failure (44).

The local microenvironment, including cytokines and apoptotic cells, influences macrophage populations and functions. Macrophages in patients with MI exhibit high plasticity, enabling them to regulate their functions according to the signals they receive. This plasticity helps resolve inflammation and activate tissue repair. Research has revealed the vital function of metabolic reprogramming in macrophages during this shift, suggesting that targeting metabolic pathways may represent a new treatment modality for cardiac repair following MI (43).

### 4.2 Regulation of macrophages in heart failure

Macrophages can exhibit different phenotypes, which affects the progression of heart failure. Following MI, CCR2^+^ monocyte-derived macrophages executing pro-inflammatory functional programs are associated with the inflammatory response and may worsen tissue damage; however, reparative macrophages participate in tissue repair and help resolve inflammation. Hence, the balance between different subtypes of macrophages plays an important role. In this regard, an imbalance—particularly an increase in pro-inflammatory macrophages—can worsen cardiac fibrosis and adverse remodeling of the heart, promoting the progression of heart failure (45).

Abnormal regulation of macrophage subpopulations is increasingly recognized as a key factor in the progression of heart failure. Studies have shown that an excess of pro-inflammatory macrophages can lead to both persistent inflammation and tissue damage. Yet a lack of reparative macrophages can slow down the healing process after cardiac injury (46). As a result, regulation of macrophage polarization has been identified as a potential strategy for improving patients’ prognosis following heart failure. Moreover, targeting aimed at promoting reparative macrophage polarization or blocking pro-inflammatory activation has demonstrated promise in preclinical models, suggesting that macrophage activity may reduce the adverse effects of heart failure (47). Approaches that enhance the shift from pro-inflammatory phenotypes to reparative macrophage phenotypes can improve cardiac function and reduce fibrosis (48). Thus, when targeting key transcription factors that regulate macrophage polarization, researchers aim to advance a more reparative phenotype, thereby promoting cardiac tissue repair and functional recovery.

### 4.3 Immune checkpoint inhibitor-associated myocarditis

The class of ICIs includes antibodies against programmed death receptor-1 (PD-1), programmed death ligand-1 (PD-L1), and cytotoxic T lymphocyte-associated protein-4 (CTLA-4) (49). By eliminating immunosuppression in the tumor microenvironment, the T-cell-mediated anti-tumor immune response can be reactivated (50). Recent single-cell studies have confirmed that the expansion of CCR2^+^ mononuclear-derived macrophages in the myocardium represents a key pathological feature (51). ICIs overcome the inhibition of T-cells by blocking immune checkpoint signaling pathways such as PD-1/PD-L1 or CTLA-4, resulting in activated T-cells recognizing and attacking autoantigens in myocardial tissue (52). Yet activated T-cells and macrophages release a large amount of pro-inflammatory cytokines, such as tumor necrosis factor -α (TNF-α), interleukin-6 (IL-6), and interferon- gamma (IFN-γ) (53). IgG deposition has been detected in the myocardial tissue of patients with myocarditis, which implies that ICI-related myocardial injury is accompanied by humoral immune activation and an autoantigen-specific antibody response. This suggests that B-cells and humoral immune mechanisms play a role in the occurrence of disease (53).

ICIs have revolutionized cancer treatment by strengthening the immune system’s ability to recognize and destroy tumor cells, although their use is associated with the possibility of a severe adverse event—namely, ICI-related myocarditis. This adverse event is marked by the inflammatory expansion of CCR2^+^ MDMs in the myocardium, which can lead to significant cardiac dysfunction and increased mortality (54, 55). The presence of inflammatory macrophages is a key aspect of the pathophysiology of ICI-associated myocarditis, given that they are important in mediating the immune response against cardiac tissues. The activation of these cells is often driven by the interaction between CD8+ T-cells and macrophages promoted by the IFN-γ signaling pathway. This interaction enhances both recruitment and activation of macrophages, and it also exacerbates the inflammatory response, leading to myocardial dysfunction (54). Therefore, patients who are treated with ICIs, particularly as part of combination therapies, should be closely monitored for signs of myocarditis. In particular, symptoms such as arrhythmias and heart failure may develop acutely or subacutely after the start of treatment (48).

### 4.4 The relationship between macrophages and cardiac fibrosis

Macrophage activation in response to cardiac injury triggers a series of inflammatory responses. The cells release cytokines and growth factors, which drive fibroblast activation and differentiation into myofibroblasts—that is, the key effector cells involved in fibrosis (56). This process is vital for the structural remodeling of the heart following injury; however, when dysregulated, it can lead to excessive fibrosis and heart failure. Recent studies have elucidated the heterogeneity of macrophage populations, which include both embryonic-derived CCR2^-^ resident and CCR2^+^ monocyte-derived recruited macrophages. Each subset plays a unique role in the fibrotic process.

The role of resident macrophages in fibrosis depends on the context. During the early repair stage following MI, embryonic-derived resident macrophages (e.g., the CCR2^-^ and LYVE1^+^ subsets) can inhibit fibroblast activation, promote angiogenesis, and limit fibrosis. However, under advanced or chronic inflammatory conditions, these resident macrophages may be reprogrammed by the microenvironment to perform pro-fibrotic functions. CCR2^+^ monocyte-derived recruited macrophages are usually pro-inflammatory and pro-fibrotic during the acute inflammatory phase. These functions reflect the dynamic characteristics of macrophage biology and therefore emphasize the importance of subgroup-specific and time-resolved analyses. For example, resident cardiac macrophages can suppress fibrosis and stimulate angiogenesis, whereas recruited macrophages often worsen cardiac fibrosis (57).

As macrophages drive cardiac fibrosis via specific signaling pathways and molecular mechanisms, researchers can develop strategies to regulate these pathways and mechanisms by elucidating the related processes. For example, inhibiting the transforming growth factor-beta (TGF-β) signaling pathway or blocking specific macrophage activation signals could effectively reduce fibrosis and improve cardiac function (58).

### 4.5 The role of macrophages in cardiac electrophysiology and arrhythmias

Cardiac macrophages play a dual and opposing role in electrophysiology, maintaining homeostasis conduction and driving pathological arrhythmias. Embryonic-derived CCR2^-^ resident macrophages support homeostatic conduction. Indeed, the landmark primary study by Hulsmans et al. revealed that a specific resident cardiac macrophage subpopulation (TIMD4^+^, CCR2^-^) can promote atrioventricular node conduction (59). More specifically, these macrophages form gap junctions with cardiomyocytes via connexin-43 (Cx43) and achieve electrical coupling. Thus, gene knockout of these cells in mice leads to atrioventricular block, which highlights their important role in maintaining normal heart rhythm (59).

CCR2^+^ monocyte-derived recruited inflammatory macrophages drive pathological arrhythmias, and a recent study by Hulsmans et al. identified a different pathogenic mechanism in this regard (60). In an atrial fibrillation model, the recruited CCR2^+^ monocytes differentiate into SPP1^+^ (osteopontin) macrophages and infiltrate the atrial myocardium. While these macrophages do not support conduction, they may induce atrial fibrillation by promoting a pro-fibrotic and pro-inflammatory microenvironment and changing the electrophysiological properties (60).

The net effect of these two functions on cardiac electrophysiology depends on the balance between the following: (1) tissue-resident CCR2^-^ macrophages that provide steady-state electrical coupling and (2) recruited CCR2^+^/SPP1^+^ macrophages that drive pathological remodels and arrhythmias (**Figure 2**).

**Figure 2:**
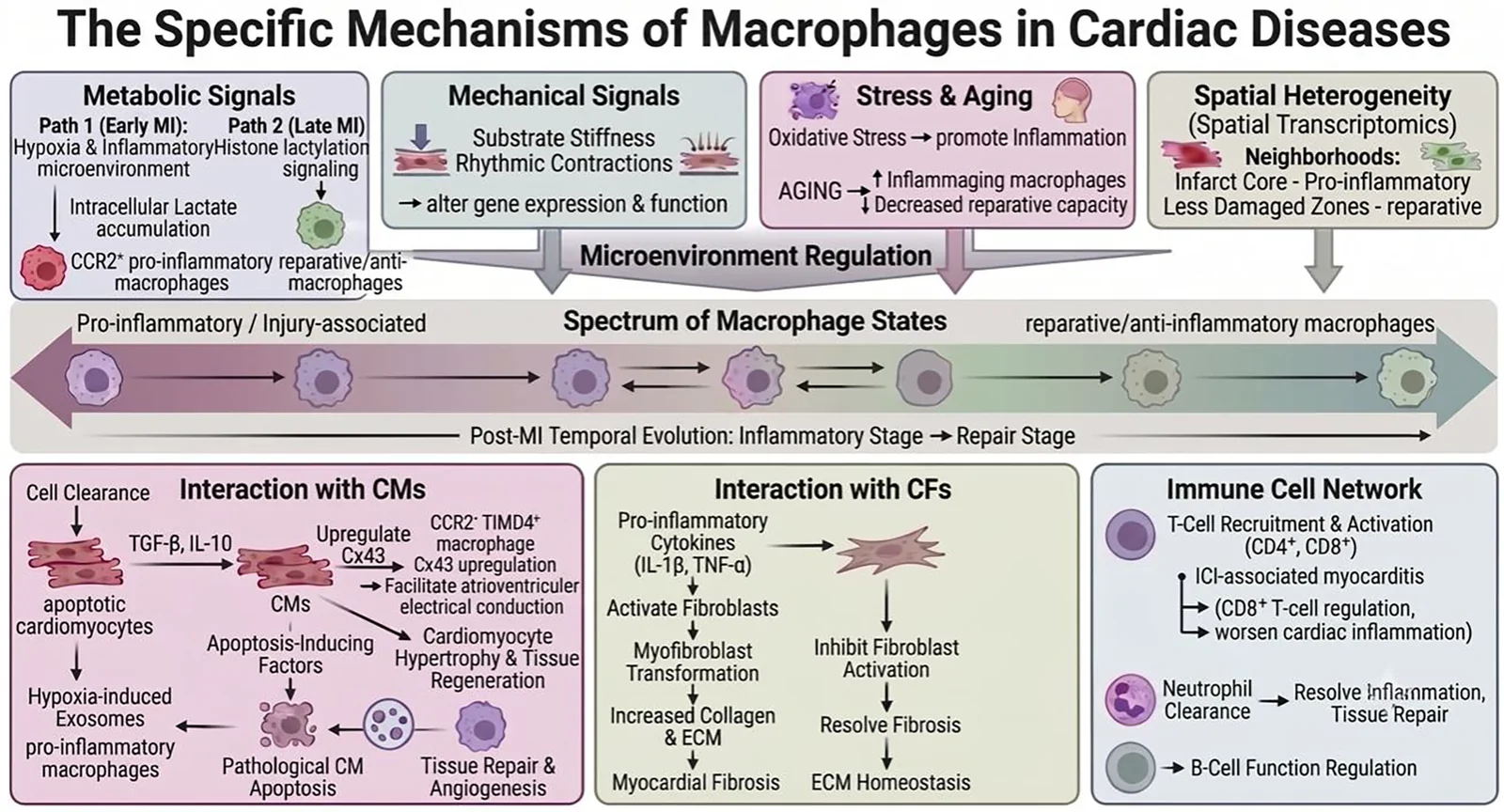
The specific mechanisms of macrophages in cardiac diseases.

## 5 Intercellular communication and microenvironment regulation of cardiac macrophages

### 5.1 Interaction between macrophages and cardiomyocytes

Macrophages secrete cytokines and metabolic products in order to regulate the cellular environment and drive tissue repair. In this way, they impact cardiomyocyte function and survival. For example, macrophages secrete TGF-β and interleukin-10, which have been shown to upregulate Cx43 expression in cardiomyocytes, thereby enhancing intercellular communication and electrical conduction (61). Additionally, macrophages participate in the clearance of apoptotic cardiomyocytes and the regulation of inflammatory responses. Both processes are essential for the resolution of injury and the promotion of tissue repair. For example, macrophages with reparative functions are associated with reparative responses and linked to improved cardiac function following MI due to driving the transition from inflammation to tissue repair (62).

Recent studies have emphasized the role of cardiomyocyte-derived exosomes. These exosomes mediate communication with macrophages, especially under hypoxia conditions, and can modulate macrophage polarization, influencing their phenotype toward a reparative state (63). Conversely, macrophage-derived factors can induce cardiomyocyte apoptosis under pathological conditions. These findings highlight the dual functions of macrophages in relation to cardiac health and disease (64).

The interaction between macrophages and cardiomyocytes is involved in the regulation of ECM remodeling. This process is important for cardiac repair after injury. Macrophages are vital for ECM remodeling due to secreting matrix metalloproteinases and other factors. These molecules trigger the migration of cardiomyocytes to injured areas and drive tissue regeneration (65). The metabolic state of macrophages governs their interactions with cardiomyocytes. Thus, pro-inflammatory macrophages may worsen cardiac injury, whereas reparative macrophages promote healing and tissue repair (66).

### 5.2 Synergistic regulation of macrophages and cardiac fibroblasts

Macrophages can regulate the proliferation and collagen synthesis of cardiac fibroblasts. In so doing, they influence the development of myocardial fibrosis. Interestingly, the distance between these two types of cells in a healthy heart is relatively far. Yet transcriptomic analyses have revealed that following MI, macrophages and myofibroblasts in the core area of the infarction closely coexist (67). The polarization of macrophages also exhibits a temporal transition after MI. In the early inflammatory stage, a large number of classic pro-inflammatory-activated macrophages are recruited to secrete pro-inflammatory mediators, such as IL-1β and TNF-α. During the repair stage, substitution-activated reparative macrophages gradually take over and secrete reparative and pro-repair factors, such as TGF-β (68). This dynamic transformation of the macrophage phenotypes is related to changes in the spatial distribution of fibroblasts, which determine the process of cardiac repair and fibrosis. This interaction is obvious in the post-ischemic heart, where macrophages may either promote or inhibit fibroblast activation according to their polarization state. Pro-inflammatory cytokines tend to enhance both fibroblast activation and collagen deposition, which contribute to fibrotic remodeling following MI (69). Reparative macrophages exhibit reparative features and can inhibit fibroblast activation and exert a protective effect against fibrosis (70). The dual functions of macrophages reflect the complex nature of cardiac tissue remodeling. They also emphasize the importance of understanding the specific signaling pathways that regulate the interactions between macrophages and fibroblasts.

For instance, studies have confirmed that embryonic-derived CCR2^-^ resident macrophages may inhibit excessive activation of fibroblasts and collagen deposition through contacting fibroblasts and secreting reparative factors such as IL-10, which maintains the homeostasis of the ECM under healthy conditions (71). Macrophages can also infiltrate cardiac tissue and drive activation of the signaling pathways in fibroblasts by secreting pro-inflammatory cytokines, such as IL-1β and TNF-α. This may promote the phenotype transformation of cardiac fibroblasts into myofibroblasts and increase the secretion of both collagen and ECM, thereby exacerbating cardiac fibrosis and causing cardiac function to deteriorate under pathological conditions (72).

Different macrophage subsets exert effects on fibroblast regulation, adding complexity to the search for the new strategies to treat cardiac fibrosis. Recent studies have shown that certain macrophage subsets with reparative functions (e.g., the M2b type) can inhibit cardiac fibroblast activation and migration, reducing fibrosis and improving cardiac function following ischemic injury (70). Macrophage subsets with that promote fibroblast activation (e.g., the M2 type) also drive collagen production. This indicates that the balance between these macrophage subtypes is important in relation to the outcome of cardiac repair (73). Furthermore, the signaling molecules released by macrophages are important when it comes to mediating fibroblast responses. IL-1β is known to enhance fibroblast activation and promote a pro-fibrotic phenotype, which aggravates cardiac fibrosis (74).

### 5.3 The interaction network of macrophages with immune cells

Macrophages are vital for initiating and maintaining immune responses and for regulating the function of T- and B-cells during cardiac inflammation. Activated macrophages produce many cytokines and chemokines. These mediators regulate T-cell differentiation and activation, thereby shaping the adaptive immune response in the heart (75). Prior studies have shown that macrophages can promote the recruitment of CD4+ T helper cells and CD8+ cytotoxic T-lymphocytes to inflamed cardiac tissues, which enhances the local immune response (76). Moreover, the interaction between macrophages and B-cells is key to antibody production. This process can help resolve inflammation, although it may lead to autoimmunity if dysregulated. Neutrophils serve as the first responders to inflammation and also interact closely with macrophages. In cases of MI, macrophages can clear apoptotic neutrophils, helping resolve inflammation and promote tissue repair (77).

In ICI-associated myocarditis, the signaling pathways between macrophages and CD8+ T-cells are particularly important. ICIs are used to enhance T-cell responses against tumors. More specifically, they can drive the activation of autoreactive T-cells that target cardiac tissue, leading to myocarditis. Here, macrophages regulate T-cell function through expression of co-stimulatory molecules and secretion of pro-inflammatory cytokines. These effects may worsen cardiac inflammation (78).

### 5.4 The influence of the microenvironment on macrophage function

The cardiac microenvironment is important when it comes to shaping the functional state of macrophages. These cells are key to both cardiac homeostasis and the pathological processes of heart disease. Factors such as the local metabolic conditions, oxidative stress, and mechanical stress may regulate macrophage polarization and functionality. In addition, the cardiac metabolic environment determines whether macrophages adopt a pro-inflammatory or reparative phenotype. Increased substrate stiffness shifts macrophage activation, a process marked by increased expression of pro-inflammatory factors and cytokines, including nitric oxide synthase (79). Oxidative stress is common in cardiac disease and can boost inflammation in macrophages and therefore complicate cardiac healing following MI (80).

Composition of the ECM and certain metabolic byproducts also play key roles in regulating the fate of macrophages. Metabolites such as lactate and adenosine can regulate macrophage polarization by driving macrophages into a reparative state that aids tissue recovery (81). Such metabolic reprogramming is key to enabling macrophages to shift from inflammation to tissue repair. Following MI, local secretion of cytokines and growth factors regulates macrophage function and drives a shift toward a reparative phenotype that is beneficial for cardiac repair (82).

Mechanical stress is a common feature of cardiac disease and can impact macrophage function. For instance, the mechanical environment of the heart, such as rhythmic contractions and different pressures, influences macrophage morphology and function. Mechanical forces can also modify macrophage gene expression patterns, which change inflammatory responses and affect tissue repair (83).

### 5.5 Spatial transcriptomics reveals macrophage cell neighborhoods

Recent advances in spatial transcriptomics have deepened understanding of the distribution and interactions of macrophages in different anatomical areas of the heart. In particular, this new technology allows for high-resolution analysis of cellular composition and gene expression, thereby providing valuable insights into the spatial structure of immune cells. To date, spatial transcriptomics studies have revealed the specific macrophage populations that inhabit distinct cardiac regions. Each population has a characteristic transcriptional profile associated with its functions in relation to health and disease (16).

The identification of macrophage cell neighborhoods by means of spatial transcriptomics helps clarify the disease-specific functions of these cells. In patients with MI, the macrophages located near damaged cardiomyocytes show pro-inflammatory expression patterns, while those in less affected areas may exhibit reparative phenotypes (84).

### 5.6 The role of macrophages in cardiac aging

Macrophages are vital for maintaining tissue integrity and responding to injury, although their functional phenotype changes during aging. This shift helps increase the availability of pro-inflammatory macrophages, which are associated with chronic inflammation and tissue damage, while the number and function of reparative macrophages, which are important for tissue healing and regeneration, decline as the heart ages. This imbalance in terms of macrophage populations impairs the ability to respond to stress and injury in the heart, leading to a decline in cardiac function and an increase in the risk of developing age-related cardiac diseases (85).

Cardiac macrophages are part of a complex multicellular communication network. More specifically, their interactions with cardiomyocytes, fibroblasts, and immune cells are coordinated by shared microenvironment signals (86). This integrated network determines the outcome of tissue repair and fibrosis—that is, dysfunction or arrhythmia. A key challenge entails understanding how these dynamic interactions evolve during the disease process and how to regulate them without disrupting homeostasis of the heart (**Figure 3**).

**Figure 3:**
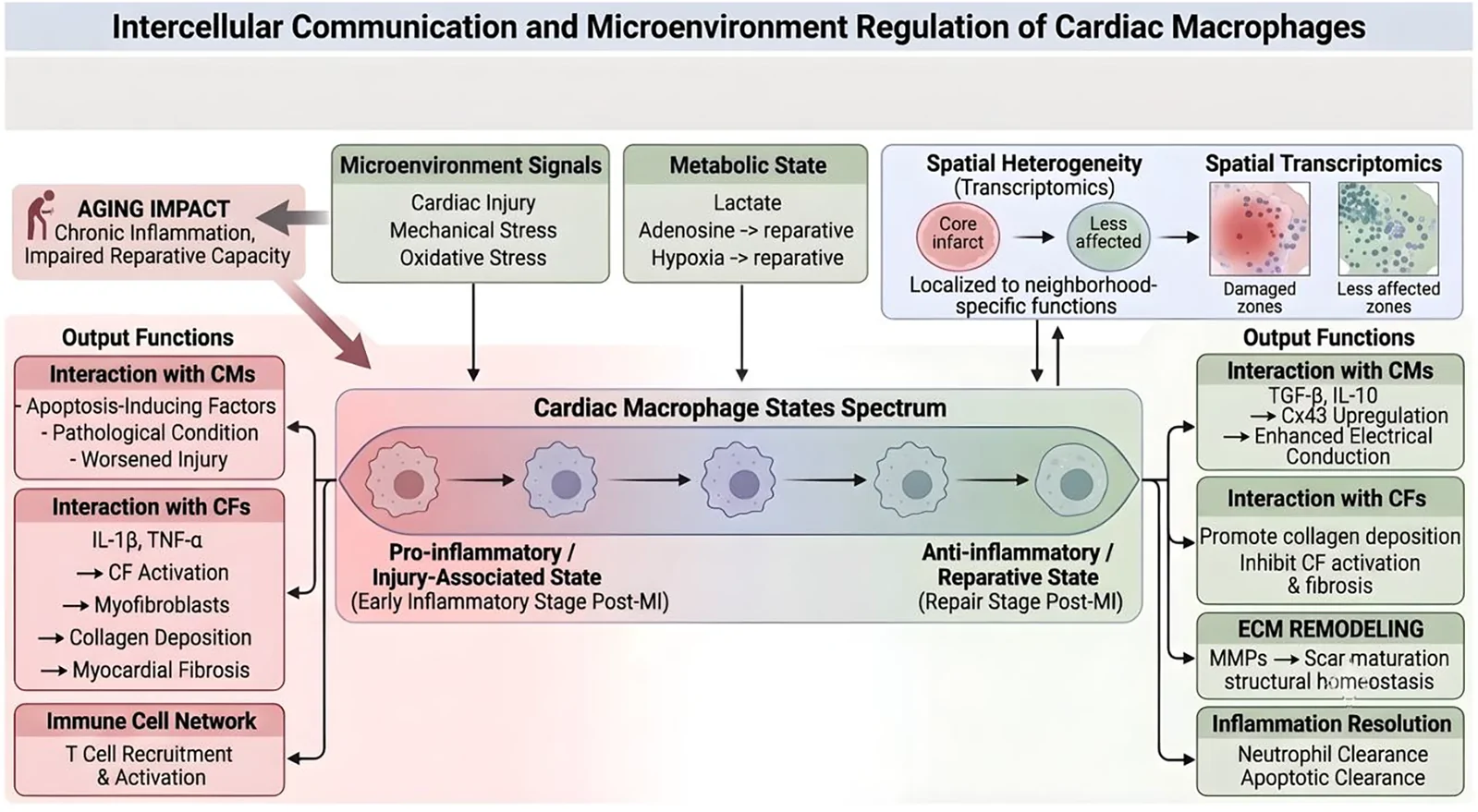
Intercellular communication and microenvironment regulation of cardiac macrophages.

## 6 Macrophage-based therapeutic strategies and future perspectives

### 6.1 Therapeutic approaches targeting macrophage polarization regulation

The targeting of macrophage polarization represents a promising strategy for the treatment of cardiac disease. Indeed, promoting reparative macrophage states and inhibiting excessive pro-inflammatory macrophage function can aid cardiac repair and reduce inflammatory damage. This suggests a potential direction for therapeutic development (87). Small molecules, antibodies, and gene-editing techniques are all currently being explored with regard to regulating macrophage polarization. Use of specific inhibitor pathways, such as the NF-κB signaling cascade, can reduce the overall inflammatory microenvironment associated with cardiac disease (88).

Of course, it must be acknowledged that there are significant species-related differences between mouse and human cardiac macrophages. In mice, the resident macrophages are mainly self-sustaining and originate from embryonic progenitor cells (13). In contrast, human cardiac macrophages have a higher renewal rate and are more dependent on continuous monocyte recruitment. The marker profiles and functional responses of macrophage subsets may also differ among species.

In addition to small molecules, mesenchymal stem cells have been identified as regulators of macrophage polarization. They can secrete different factors that drive macrophages toward a reparative phenotype, helping with tissue repair and reducing inflammation (89). Progress in terms of gene intervention technologies such as CRISPR/Cas9 has enabled precise modifications of macrophage polarization pathways, allowing researchers to explore new strategies to improve the outcomes of patients with cardiac disease (90).

The use of exosomes derived from macrophages also represents a new therapeutic strategy. These vesicles carry proteins, lipids, and RNAs that can regulate macrophage behavior and drive toward a reparative phenotype, thereby enhancing tissue repair (91). This method uses the natural signaling pathways of macrophages and offers a targeted delivery system for therapeutic drugs.

Nevertheless, while the above-mentioned therapeutic strategies for addressing macrophage polarization appear promising, there remain some limitations. The classic binary classification of macrophage activation states implies a continuous spectrum of macrophage states *in vivo*. Future therapeutic development should proceed under the guidance of spatial transcriptomics and regulate subpopulations within specific cellular neighborhoods. A combined strategy that targets both metabolic pathways and intercellular signals (e.g., TGF-β and IFN-γ) may prove more effective.

### 6.2 Metabolic regulation as a therapeutic target

Metabolic pathways offer a new therapeutic direction in terms of cardiac disease management, especially through the regulation of macrophage function. Targeting metabolic pathways such as glycolysis inhibition or oxidative phosphorylation regulation could improve both macrophage functionality and cardiac health. Moreover, metabolic reprogramming of macrophages from pro-inflammatory to reparative is important for tissue repair and resolution of inflammation following cardiac injury (48). In this regard, drugs that target specific metabolic pathways have demonstrated efficacy in preclinical models. For example, the use of alpha-ketoglutarate restores the TCA cycle and drives reparative macrophage polarization, which helps reduce cardiac dysfunction following MI (92).

Furthermore, the role of metabolic byproducts in regulating macrophage function is also gaining increasing attention. Metabolites such as lactate, succinate, and fatty acids can regulate macrophage polarization and activity. Lactate is particularly important in relation to promotion of reparative macrophage polarization, which is useful for both tissue repair and reparative responses (93).

Preclinical studies have also explored the effects of different metabolic modulators on macrophage functions and their implications for cardiovascular health. Natural products such as flavonoids and other phytochemicals can impact macrophage metabolism and function, offering a dual approach to controlling inflammation and metabolic disorders in patients with cardiac diseases (94). These compounds often act by altering key metabolic pathways, enhancing the reparative functions of macrophages and reducing pro-inflammatory cytokine production.

### 6.3 Intervention of immune checkpoint signaling pathways

ICI-related myocarditis involves a severe inflammatory response in the heart, mainly driven by the infiltration of T-lymphocytes and macrophages. This condition can lead to significant cardiac dysfunction and even mortality. Recent studies indicate that IFN-γ-driven expansion of inflammatory macrophages is important in relation to the pathogenesis of ICI-related myocarditis (54). Interestingly, in murine models of ICI-related myocarditis, blockade of IFN-γ signaling reduces the number of inflammatory macrophages and improves cardiac function (54).

In addition to the blockade of IFN-γ signaling, immunomodulatory agents offer a promising strategy for relieving cardiac inflammation. In fact, use of immune modulators such as regulatory T-cell therapies and cytokine-targeted approaches has potential in terms of reshaping the inflammatory environment in the heart during ICI therapy (95). Therapies that drive macrophages to shift from the pro-inflammatory phenotype to the reparative phenotype can benefit cardiac tissue repair and reduce inflammation. This shift is crucial, given that reparative macrophages can heal tissue and help resolve inflammation, which can counteract ICI-related cardiac inflammation (96).

### 6.4 Applications of stem cell and organoid models in macrophage research

Human heart macrophage organoids (hHMAs) have transformed both disease modeling and drug screening, offering a new way to study the complex interactions between macrophages and cardiac tissues. These organoids are generated from human pluripotent stem cells and replicate the cardiac physiological environment, similar to TRMs. This system allows researchers to explore macrophage functions in the case of MI, heart failure, and other cardiac disorders. Studies using hHMAs have demonstrated that these organoids can replicate inflammatory responses, facilitating examination of macrophages’ effects on cardiac repair and remodeling (97). The development of hHMAs has enhanced precision medicine and supported the development of individualized treatments. Moreover, patient-specific stem-cell-derived organoids allow researchers to examine the efficacy of such treatments in a controlled environment.

### 6.5 Future research directions and challenges

Exploration of macrophages’ heterogeneity and their responses to different cardiac conditions represents a critical direction for future research. Cardiac macrophages can be classified into different subsets according to their developmental origins, and each subset has specific functions in terms of cardiac health and disease (16). Future studies should focus on the changes that occur in macrophage subsets in order to elucidate how these cells alter their functions in response to environmental signals.

Furthermore, combining multi-omics approaches (e.g., transcriptomics, proteomics, metabolomics, spatial omics) may deepen understanding of cardiac macrophage biology, thereby elucidating the interactions that occur between macrophages and other cells and revealing the signaling pathways that regulate macrophage function (98).

Nevertheless, while some strategies targeting macrophages have been proposed, their transformation potential varies. As a consequence, these methods must be assessed based on prioritization, clinical reality, and related risks. The following order of priority is proposed based on the currently available evidence. Spatial transcriptomic-guided subpopulation targeting has the highest priority, while direct targeting of disease-specific macrophage subpopulations has the highest precision and the least off-target effect. When it comes to metabolic reprogramming, these drugs are already available in clinical practice and have shown preclinical efficacy in cardiac models. For exosome therapy, there appears to be promise in terms of targeted delivery. Stem cell and organoid models have shown good effects on disease modeling and drug selection. With regard to polarization control (pro-inflammatory toward reparative functional programs), while it is conceptually attractive, due to the oversimplified binary classification of macrophage activation states and the risk of promoting fibrosis or tumorigenesis, there remain problems in relation to its clinical implementation.

In addition, several problems must be solved prior to clinical transformation. First, species-related differences: mouse models overestimate the contribution of self-renewing resident macrophages, while human studies overly rely on monocyte recruitment. Second, delivery challenges: systemic delivery of metabolic regulators may lead to off-target metabolic effects. Third, biomarker development: non-invasive imaging or blood biomarkers are needed to monitor the dynamic changes in macrophage subsets.

Furthermore, these therapies involve specific risks. In terms of polarization control, mandatory induction of a reparative macrophage phenotype may promote fibrosis, drive tumor growth, or increase infection. When it comes to metabolic targeting, long-term inhibition of glycolysis may impair normal immune surveillance. Stem cell therapy poses the risks of teratoma formation, immune rejection, and arrhythmia increase. Moreover, there are unknown effects associated with long-term exosome administration.

## 7 Conclusion

Cardiac macrophages represent a complex and vital part of the heart’s immune system. They play a dual role in promoting inflammation and tissue repair, which emphasizes the need for a refined approach to cardiac macrophages in research and therapy. Immunomodulatory approaches that target cardiac macrophages show significant promise and may slow disease progression and improve clinical outcomes. Still, joint efforts by scientists, clinicians, and bioengineers are required to develop better methods.

Prior studies of cardiac macrophages have generated deeper understanding of their dual and dynamic functions in both health and disease. Additional research and clinical applications are required to explore the therapeutic value of cardiac macrophages and improve patients’ prognosis.
